# Parturition in Mammals: Animal Models, Pain and Distress

**DOI:** 10.3390/ani11102960

**Published:** 2021-10-14

**Authors:** Julio Martínez-Burnes, Ramon Muns, Hugo Barrios-García, Dina Villanueva-García, Adriana Domínguez-Oliva, Daniel Mota-Rojas

**Affiliations:** 1 Animal Health Group, Facultad de Medicina Veterinaria y Zootecnia, Universidad Autónoma de Tamaulipas, Victoria City 87000, Tamaulipas, Mexico; hbarrios@docentes.uat.edu.mx; 2 Agri-Food and Biosciences Institute, Hillsborough, Co Down BT26 6DR, Northern Ireland, UK; rmunsvila@gmail.com; 3 Division of Neonatology, Hospital Infantil de México Federico Gómez, Mexico City 06720, Mexico; dinavg21@yahoo.com; 4 Neurophysiology, Behavior and Animal Welfare Assessment, DPAA, Universidad Autónoma Metropolitana (UAM), Unidad Xochimilco, Mexico City 04960, Mexico; mvz.freena@gmail.com

**Keywords:** parturition, delivery, whelping, farrowing, labour, pain, distress

## Abstract

**Simple Summary:**

Labour is considered a painful episode where complex physiological, hormonal, morphological, and behavioural changes are present. During animal parturition, the recognition and treatment of pain is not a regular practice, although there are several consequences derived from pain in the mother and the newborn. This review discusses current knowledge about human labour pain, the relevant rat model’s contribution to human labour pain, and model parturition pain mechanisms in small and large animals. Parturition’s pain represents a potential welfare concern; therefore, pain indicators and appropriate analgesic therapy are also analyzed in this work including the relevance of analgesics and the welfare implications of pain during this physiological stage.

**Abstract:**

Parturition is a complex physiological process and involves many hormonal, morphological, physiological, and behavioural changes. Labour is a crucial moment for numerous species and is usually the most painful experience in females. Contrary to the extensive research in humans, there are limited pain studies associated with the birth process in domestic animals. Nonetheless, awareness of parturition has increased among the public, owners, and the scientific community during recent years. Dystocia is a significant factor that increases the level of parturition pain. It is considered less common in polytocous species because newborns’ number and small size might lead to the belief that the parturition process is less painful than in monotocous animal species and humans. This review aims to provide elements of the current knowledge about human labour pain (monotocous species), the relevant contribution of the rat model to human labour pain, and the current clinical and experimental knowledge of parturition pain mechanisms in domestic animals that support the fact that domestic polytocous species also experience pain. Moreover, both for women and domestic animal species, parturition’s pain represents a potential welfare concern, and information on pain indicators and the appropriate analgesic therapy are discussed.

## 1. Introduction

Parturition is a complex process that involves changes at the hormonal, physiological, morphological, and behavioural levels [1,2]. One of the characteristics of this event is the manifestation of pain that can vary in severity and duration depending on the species studied. Parturition is a significant event for numerous species and is frequently the most painful experience that females suffer [3]. Unlike other acute pain experiences, labour pain is physiological and unique, not associated with disease or trauma, and its presence does not indicate any pathology, but the progression of labour itself [4], and the most basic experience of life, the birthing of a new individual [5]. However, several factors may hinder the normal process of parturition and modify or increase the degree of pain caused by it.

The International Association for the Study of Pain (IASP) defined pain “as an unpleasant sensory and emotional experience usually associated with tissue damage or described in terms of such damage” [6,7,8], the definition had remained unchanged since 1979. However, in recent years, advances in our understanding of pain in its broadest sense warranted a re-evaluation of the definition [9,10]. Recently in 2019 [11], the current definition was revised to “an unpleasant sensory and emotional experience associated with or resembling that associated with, actual or potential tissue damage”. Also, with the following notes: Pain is always an individual experience modified by various degrees by biological, psychological, and social factors, through their life experiences, individuals learn the concept of pain, although pain usually serves an adaptive role, it may have adverse effects on function and social and psychological well-being, and verbal description is only one of several behaviours to manifest pain; incapacity to communicate does not negate the possibility that a human or a nonhuman animal experiences pain. A relevant update is that the pain definition should be used in humans and non-human animals [12].

A large proportion of women describe parturition as severely painful [13,14]; for that reason, labour pain in women has received great scientific interest. Research in the rat model has contributed to elucidate the subjacent neurophysiological and neuropharmacological mechanisms of pain during labour [15].

In small animals, labour pain is generally accepted as a paradoxical and challenging issue, composed of a complex, multidimensional subjective experience involving sensory and affective (emotional) elements [16]. Also, in pigs, pain is described as a perceptual event created from information assembled by specific sensitive receptors for tissue injury, transformed by ascending and descending spinal and supraspinal mechanisms, and combined into an individual sensorial event including negatively valenced emotions [17].

In cows and sows, it is recognised that pain has a beneficial role because it is intimately related to some of the neurohumoral responses required for inflammation and can alter physiological responses, which also help the individual to handle damage [18,19]. 

The individual’s expression and response to pain have an emotional and social interpretation structured by culture [8,20] and visible painful events [21]. The pain-conscious experience challenges the specific anatomical, physiological, and/or pharmacological interpretation. Moreover, it is a subjective sensation that can be suffered even without visible harmful stimulation and can be altered by beliefs, moods, customs or habits of humans who are responsible for animals, coping ability, and by behavioural experiences, including fear, memory, and stress of the different animal species [16]. 

Contrary to the extensive research in humans, in domestic animals, there are limited pain studies associated with the birth process, reflecting little interest in the subject [19]. Most studies refer to the cascade of hormonal events, morphological and behavioural changes associated with pregnancy, labour, and lactation. Similar neuroanatomical structures associated with pain perception are shared by humans and vertebrates [22,23], including nociceptors, nociceptive pathways, and processing zones in the central nervous system (CNS). Therefore, it is recognised that pain perception in humans and other mammals is similar. Most mammal species have receptors sensitive to noxious stimuli (nociceptors) and have brains with structures analogous to the human cerebral cortex. They have nervous pathways linking nociceptors to the higher brain, and it has been observed that painkillers modify the response physiologically and behaviourally [19]. 

Nonetheless, during recent years, awareness of the labour process, its impact on welfare, health, and even economic return in domestic animals has increased among the public, owners, and the scientific community. For example, in swine, the health and welfare status of the sow have been shown to reflect her capacity to generate healthy offspring [3]. In dogs, the owners increasing emotional involvement in the birthing process of their pets, and the economic importance of purebred puppies, has produced raised interest in enhancing whelp survival. [24]. Dystocia is usually considered to be less common in polytocous species such as the pig than in monotocous animal species and humans [25]. The number of newborns and smaller size in the former might assume that the labour process is less painful. In this review, the current knowledge about human labour pain (monotocous species), the contribution of the rat model related to human labour pain, and the current clinical and experimental knowledge of parturition pain mechanisms in pigs, with physiological and anatomical similarities with humans for the study of labour pain, support that domestic polytocous species also experience pain. Both for women and domestic animal species, the pain associated with parturition represents a potential welfare concern. The above-mentioned matters justify the need to review the published information of labour pain and current advances drawing on examples from monotocous and polytocous species, based on the availability of research in humans and on pain study models in some domestic animal species.

## 2. Physiological Response to Labour: Comparative Studies

The timing of labour in humans continues to be an enigma, and more reliable knowledge of these mechanisms is essential to avoid unfavourable consequences of pregnancy. Parturition, a fundamental and species-specific component in the reproductive cycle, is under intense selection pressure. A broad diversity in gestation length and neonatal maturity at birth occurs in species unrelated to the size at birth. Human neonates are similar to other altricial species in terms of maturity at delivery, although primates are usually considered precocial at birth [26].

In humans, a natural time of parturition begins parallel to foetal organs maturity, generally 37–40 weeks gestation. Prevailing hypotheses of labour induction are mainly associated with foetal-maternal endocrine and immune alterations in utero relating to foetal development [27]. Childbirth is when a foetus and placental tissues are released from the uterus through the vagina. In women, the birth process can be divided into two stages. The first is divided into the latent and the active phase. The latent occurs when the woman undergoes regular and painful contractions until the cervix dilates four centimetres. The active one goes from a dilation of the cervix of four centimetres to full dilation. The second stage, in turn, is subdivided into two phases; the descending in which the baby’s head drops towards the pelvic floor, and the expulsion when the dam is actively expelling the child [28].

Labour monitoring helps determine its stage and progress, and multiple modalities are used. Consecutive cervical exams are helpful to delimit cervical dilation, effacement, and foetal position, also called tocography. Cardiotocography is used to monitor the foetal heart frequency and capacity of uterine contractions. Also, it is helpful to assess foetal well-being throughout labour continuously. Physicians determine the patient’s stage of parturition and monitor the progression of labour based on information from monitoring and cervical examinations [29,30]. 

In female Dorper sheep, the use of echocardiography has been implemented for accurate readings from the third month of gestation, in which foetal cardiac monitoring allows the identification of congenital or developmental anomalies, and hypoxemia and acidemia due to placental dysfunctions that compromise the viability of the foetus, something that, in production species, especially reproducers, is essential [29,30]. Similarly, foetal abnormalities in horses are identified using the biophysical profile, considered the gold standard in reproduction to assess foetal well-being by determining the foetal heart rate, the diameter of the aorta and the thickness of the uteroplacental unit [29,30]. 

Diagnosis of labour initiation is a challenging and essential decision for those who provide maternity care. The initial stage of labour, through active uterine contractions, accomplishes the goal of reducing, dilating, or opening the cervix by at least 10 cm in diameter to permit the foetus to pass from the uterus to the vagina. The duration of both stages of labour are clinically significant and hence need uniform strategies to measure. An extended latent phase of parturition is linked with a higher risk for oxytocin increment of labour, caesarean section, amniotic fluid stained with meconium, an Apgar score less than seven at 5 min, demand for newborn resuscitation and ingress to the neonatal intensive care unit (NICU) [30,31,32,33]. There is a significant discrepancy in the definitions of labour initiation in the research reports. Still, there was little consensus between studies referring to the same type of parturition initiation (e.g., active labour phase) and an indication of labour initiation, except that 100% of the definitions of latent labour phase refer to the presence of frequent painful contractions [34].

Based on the essential nature of parturition, there is a lack of understanding of how the way by which a baby is born can have later physiological consequences for newborns. More comprehensive knowledge of how the form, start, sequence, and duration of parturition can influence healthy development and long-term well-being consequences for the infant is crucial because of the global increase in delivery by caesarean section (CS). In 2595 women subjected to CS (50.5% of the total sample), a retrospective study made by Khasawneh et al. [35] found that fetal distress was the main indication of CS (15.5%). From the delivered newborn, 16% required hospitalization due to transient tachypnea and respiratory disease syndrome. The increase in CS is also in domestic animals, particularly in some breeds of dogs, and the Belgian White and Blue cattle [36]. The principal mechanisms associated with explaining why the mode of parturition, spontaneous or induced/increased vaginal birth versus CS, may alter neonate development include (1) physical stress and exposure to stress hormones during parturition, (2) unusual bacterial colonisation of the child’s intestine, and (3) epigenetic alteration of gene expression [37]. 

Additionally, the risk of CS in the newborn includes immune deficits (i.e., asthma) and alterations of the young microbiome [38]. Ensuring normal and safe physiological conditions could evade adverse consequences of the mode of parturition. Assessment, care plan, health training, and improvement of natural processes could establish the framework to increase the capacities of women in preparation for childbirth and maternity [39]. 

## 3. Labour Pain (Parturition Pain Stimuli)

Recently, foetal tissue senescence was reported in association with foetal growth causing sterile inflammatory markers that can disseminate as paracrine foetal signals linked with childbirth. Homeostatic irregularities created by endocrine and paracrine constituents generate an inflammatory overload that interferes with the preservation of gestation. These signals transform the inactive myometrium into the active state. Although, characteristics of the foetal or maternal-derived signals and their specific mechanism in initiating delivery remain unclear [40]. More recently, Menon (2019) reported that foetal membrane senescence and the associated inflammation function as a paracrine signalling system during labour [41].

Labour is a dynamic process of delivering a foetus and is characterised by regular, painful uterine contractions that progress in number and severity. Labour pain has visceral and somatic components. The cervix has a primary function in both the first and second stages of delivery. Parturition pain occurs out of a range of physiological factors like uterine contractions and cervical dilation, accompanied by mental and emotional constituents, including fear and anxiety. Other constituents such as maternal age, parity, the physical health of women in labour, and the maternal situation can also influence its severity and duration [34,39]. The behaviours displayed by women in parturition can also induce labour pain and can be associated with the length of the stages and the severity of the pain. 

Consequently, it is suggested to recognise this problem simultaneously with physiological signs and clinical tests for better management of women in labour [42]. Based on the several similarities among animals and humans in anatomical and chemical pathways of pain perception, it is commonly admitted that pain perception is comparable in humans and other mammals. Hence, from the dam perspective, parturition in all species is usually admitted as a painful process. Broadly, childbirth associated with difficult parturitions or dystocia may produce unacceptably severe pain levels in the dam. Thus, for example, in sows and cows, it is recognized that pain has a beneficial role because it is closely related to some of the neurohumoral responses required by inflammation, which can alter physiological responses and help the individual to manage the damage [18]. In pigs, as it is in other species, parturition is expected to be painful, and pain probably originates from uterine contractions, piglet ejection, and uterine inflammation due to a piglet litter delivery [18]. 

Regarding inflammation, during parturition, there are notable alterations in the acute-phase protein concentrations. Haptoglobin (Hp) and Serum Amyloid A levels rise throughout calving, higher in heifers than in pluriparous cows, implying a higher inflammation or trauma around calving, producing higher pain levels [18]. In pigs, C-reactive protein (CRP) and Hp are recognised as the most reliable markers of inflammatory damage, and high levels have been described in sows one week after farrowing [43]. Furthermore, higher Hp values are described in primiparous sows than pluriparous sows [44]. Kostro et al. [45] reported inflammation of the reproductive tract and mammary glands associated with raised levels of CRP in sows with Mastitis, Metritis, and Agalactia (MMA).

Primarily, in the lower uterine segment, the concentrations of interleukin (IL) 1β, 6, and 8 were found to be significantly higher when the cervical dilatation was 4 cm (6.6, 67.7, and 125.8 pg/mg, respectively) and promoted normal and premature labour in humans [46]. Another study compared the presence of cytokines in the uterine segment and the amniotic fluid in patients undergoing CS. The authors found that IL-6 concentrations in the amniotic fluid increased earlier (at 2–3.9 cm) than IL-8 (at 6 cm), and no correlation was found in the uterus for this cytokine [47]. During the labour term and preterm, the increase in other cells, such as adhesion molecules in the lower segment and cervix, have also been reported to trigger the cervical tissue ripening [48]. Myometrial inflammation is also involved in the labour beginning, suppressing progesterone action [49], driving pro-labour gene expression, including prostaglandin biosynthetic enzymes [50] and the oxytocin receptor [51]. Studies emphasize the relationship between labour and inflammation, confirming a severe inflammatory myometrial recruitment of monocytes, neutrophils, and macrophages [52,53,54]. In more recent publications, the same group demonstrated that inflammatory cytokines, bacterial lipopolysaccharide (LPS), and monocytes could increase myometrial cell contractions, the first by raised prostaglandin synthesis and the second for a direct effect on ROCK [55,56]. Therefore, this is compatible with a common antagonist relationship among progesterone and inflammation, which is the essence of diverse hypotheses for the labour start [49,57]. One of the proposed mechanisms includes the secretion by the mature foetal lung of surfactant protein A (SP-A), which initiates the proinflammatory transcription factor NFκB [58]. The second mechanism includes myometrial stretch, whereby the growing pregnancy rises myometrial wall tension, stimulating the proinflammatory transcription factors NFκB and AP-1, which induce the proinflammatory cytokines expression [59]. Although, some key question remains; does myometrial inflammation occur before the onset of labour, or is it merely a consequence of labour? Interestingly, Singh et al. [60] studied myometrial samples from women at various pregnancy and spontaneous labour stages, evaluating some proinflammatory factors and inflammatory cells, and the authors suggested that myometrial inflammation is a consequence rather than a cause of term labour [60].

## 4. Origin and Transmission of Parturition Pain Stimuli

Parturition pain is a deeply personal, challenging, sensitive, and meaningful experience, very different from other types of distress. In humans, the main determining and influencing factors in labour pain are cognitive, social, and environmental. Interestingly, if a mother can maintain the feeling that her pain has a goal (i.e., her body working for childbirth), if she translates her pain as productive (i.e., a process for the desired goal), along with secure and supportive delivery conditions, she would be expected to undergo pain as a life-changing and non-threatening event. Transforming the conceptualisation of labour pain to a beneficial and fruitful process could be a start to improving women’s experiences of it and diminishing their need for pain interventions [61].

During the stages of the labour process (preparation of the uterus, active labour, or the expulsion phase and delivery or expulsion of the placenta), a series of endocrine events have been triggered that mark the anatomical changes to promote the birth [18]. The preparation of the uterus and cervix for birth is characterised by subclinical myometrial contractions, progressive dilation of the cervix, and the positioning of the foetus for expulsion [62,63]. It is worth noting that uterine contractions are weak but regular [15]. Initial preparation of the birth canal takes place along with the usual prepartum endocrine alterations (relaxin discharge, a decrease of progesterone and increased oestrogen and prostaglandin synthesis) [29,64,65]. In pigs, oestrogens and relaxin cause cervix distension by altering the action of collagen [65,66]. Cervical softening and ripening is also influenced by IL-8, IL-6, and granulocyte colony-stimulating factor for remodelling the cervical tissue and opening the cervical canal [67]. Chemokines and prostaglandins in the amniotic fluid and cervix, and the release of collagenases by the neutrophils, which increase together with macrophages in the lower uterine segment, contribute to the cervical dilatation in the prepartum phase [68]. However, it is important to recognize the differences between species; for example, in equine species, maternal progesterone levels during the late stage of gestation are almost nil, and the production of progesterone and estrogens depends on the foetoplacental unit and steroid precursors formed by the foetus. This event only occurs in mares [69,70,71].

**Figure 1:**
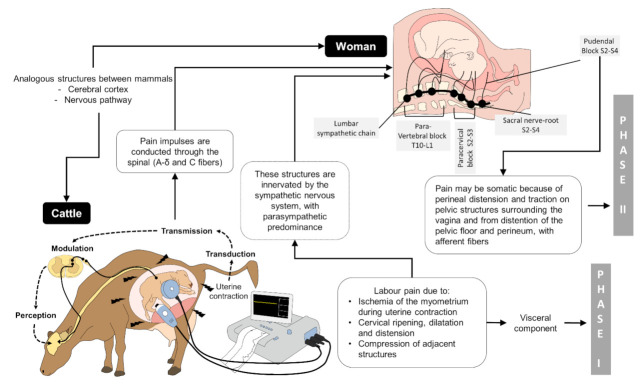
Pathway of pain in labour. In the first stage, there are slight but constant uterine contractions; as the strength of the contractions increases, concomitantly with the distension, effort, and tear of the lower uterine segment and the cervix, it becomes strongest and induces visceral pain with afferent information travelling within the hypogastric and pelvic nerves [15]. In the second labour stage, the expulsion phase is described as the most painful stage due to cervix distension and pressure on the pelvis and perineum, with pudendal nerve innervation. The nociceptive stimuli are processed and transmitted at the dorsal horn of the spinal cord, via the spinothalamic region to the thalamus, brain stem, and cerebellum, where spatial and temporal analysis take place, and also to the hypothalamic and limbic systems [5]. The third stage of labour, the delivery, consists of the expulsion of the placenta and is not painful [86].

From a physiological point of view, labour pain in humans is induced by the ischemic myometrium while uterine contraction, cervical maturation and dilation (stretching of the vagina and perineum), and distension and compression of adjacent pelvic structures [69,70,71,72]. Myometrial contractions differ between species and also between individuals. Usually, the extent, number, and amplitude of myometrial contractions rise and become regular roughly 12 h before the start of the second stage. Eventually, the foetus responds with movements to prolonged uterine stress created by early-stage myometrial contractions [63]. Labour pain also has a visceral component during the dilation of the cervix via afferent fibres in the central nervous system. In humans, these nociceptive stimuli of the dilation stage are predominantly transmitted between spinal nerves T10 to L1 [72] (Figure 1).

Pain pulses are carried out via the spinal cord mainly by afferent A-delta and C fibres [71,73], so pain can progressively refer to the abdomen, the lumbosacral zone, the iliac crests, the gluteus, and the legs [5]. The second stage, or active labour, usually begins with the rupture of the foetal membranes and ends when the last foetus is expelled. This stage comprises terminal cervical widening, achieved by the propulsive forces of regular uterine contractions through labour [66]. Since labour progresses, distention of the maternal birth canal results in increased oxytocin discharge from the posterior pituitary, increasing myometrial contractions [63]. Oxytocin plays a vital role in the parturition progress coordinating the uterine contractions [66,74] and the foetal expulsion [65,75]. In humans, melatonin also has a supporting role in myometrial contractions. Olcese and Beesley [76] found that melatonin has a synergetic action and enhances oxytocin-induced contractions binding to specific melatonin receptors (MT2R) in the myometrium. This response also follows a circadian pattern where the labor onset is triggered during the night/morning hours. Moreover, physiological administration of the monoamine hormone induces contractibility, together with oxytocin, to induce labor [77]. Domestic animals and humans possess a high number of uterine oxytocin receptors, confirming the high sensitivity to exogenous oxytocin at the active labour stage [78,79,80,81].

Additionally, at least for rats and humans, oxytocin receptors rise to 200 times during late pregnancy compared to the non-pregnant state [82]. In humans, during the second stage of labour, pain is believed to be somatic because of the perineal distension and strain on pelvic structures surrounding the vagina and further due to the expansion of the pelvic floor and perineum, with afferent fibres between spinal nerves S2 and S4 [5] (Figure 1). It is described that the perineal stretching before spontaneous vaginal delivery is characterised by severe pain [83]. Therefore, the second stage of parturition is considered the most painful phase of labour [84]. Although practically all parturient women suffer low abdominal pain during contractions, 15% to 74% may additionally experience contraction-related low dorsum pain, sometimes continuous, including between contractions [85]. Labor and Maguire [13] point out that the severity of parturition pain intensifies as cervical dilation increases and is directly associated with strength, duration, and number of myometrial contractions. Stage III generally occurs in parallel to stage II and includes passage of the foetal membranes. In the case of polytocous species (like sows), the foetal membranes along with the foetus are regularly expelled. However, in monotocous species (like women), the complete newborn expulsion resembles the third stage. During this stage, uterine contractions remain, diminishing in amplitude but with the highest and least regular frequency [62]; this phase of labour is believed to be painless [86].

Beta-endorphin (β-end) belongs to one of three opioid peptides families, and it is implicated in regulating the body’s response to stress, including pain. Endogenous β-end has been determined around parturition and is thought to be linked to reducing pain [87], maintaining passivity [88], and regulating oxytocin release [89]. The naloxone administration diminished the nociceptive threshold of sows, but not wholly, indicating that an endogenous opioid mechanism is likely only partly involved in hypoalgesia around parturition [90].

## 5. Factors Affecting Labour Pain, Dystocia

As already presented, labour by itself is a cause of pain for any mammal female, even within physiological parameters/conditions [3]. Several factors have been identified that hinder the normal process of parturition and alter the degree of pain caused by it. Dystocia may be defined as parturition difficulty happening from extended natural parturition or extended or severe assisted extraction [19]. Dystocia translates into the inability to expel the foetus(es) through the birth canal, whether due to a physical obstacle or functional defect, associated with intolerable high levels of pain [19,91,92], and in humans, the main cause is due to foetal malposition, as reported by Hautakangas et al. [93] in 5200 women, were 296 required CS. Dystocia also leads to acute foetal distress and mortality and a decrease in the vigour of the newborn. Usually, the causes of dystocia are divided into maternal and foetal [19,62]. The former being the ones that most influence a prolonged birth in women and domestic animal species.

The maternal characteristics of prolonged labour include parity (higher pain rates in primiparous versus pluriparous women), diameter and anatomy of the pelvis, previous obstetric situations, psychological, cultural, and educational aspects. However, the environment (e.g., high temperatures) and management (inappropriate use of ecbolics and excessive obstetric manipulations) can also cause dystocia or alter the birth process [62,94]. Within foetal characteristics are higher body weight and foetal position. Intolerable pain in humans results in a high catecholamine release, which leads to less uterine blood perfusion, reduces uterine contractions’ effectiveness, and, consequently, prolongs labour [95]. Also, it induces dystocia, exhaustion, and foetal distress, in addition to postpartum posttraumatic stress disorder [5,96,97]. Sandström et al. [94] highlight the relationship between the duration (prolonged) of the second stage of labour and adverse neonatal outcomes (Table 1).

In cattle, the prevalence of dystocia is calculated between 1.5–22.6% and the leading cause of calf death at birth. It induces mastitis, and also systemic infections such as clinical metritis [98], clinical and sub-clinical endometritis in almost 51.6% of cases [99] due to placental retention in 43.1% and 37.50% of 1300 cows [100]. Barraclough et al. [101] report that one method of preventing complications during parturition is to observe the postures and activity of the animals, which usually increases by up to 80% during stage two of parturition because it is severe pain.

**Table 1:** Factors associated with dystocia, prolonged labour and pain in women.

Factor That Increases Labour Pain	References
**Maternal characteristics:** Prolonged labours Parity. The pattern of parturition pain varies among nulliparous versus pluriparous women, and it is documented that pain rates are higher in the pluriparous contrasted to the pluriparous woman. Increased “elasticity” and flexibility of the pelvic tissue in pluriparous women may decrease nociceptive stimuli during the dilation phases of labour, but stimuli increase later in childbirth due to the speed and intensity of foetal descent. The diameter and anatomy of the pelvis, length and mass, myometrium contractile forces, soft tissue stability, ejection efforts, previous obstetric/medical situations, such as hypertension or pregestational/gestational diabetes mellitus. Psychological aspects. A variety of psychological factors influence pain perception; Anxious, tense women, fear, and loneliness are associated with increased pain during labour. Cultural aspects and educational level also intervene in the perception of pain. **Foetal characteristics:** Birth weight (higher foetal body weight increased pain), Foetal occiput position/degree of flexion, and station at complete cervical dilation.	[102,103,104,105,106,107]

In female dogs, uterine inertia is the leading cause of dystocia derived from prolonged births [62,108]. Other factors related to dystocia in bitches are older age, smaller litter size, brachycephalic and achondroplastic breeds. A narrow pelvic canal can induce obstruction because of the disability of the foetus to pass normally; the obstruction due to a short pelvic channel is common in brachycephalic and achondroplastic breeds. The higher incidence of dystocia and caesarean section due to primary inertia in these breeds is considered a hereditary condition, and hence the bloodlines in which this problem is shared should be identified and breeders advised avoiding breeding from or combining such lines. Also, to use easy whelpings in their breeding programs to prevent welfare implications in the bitch and her puppies [109]. In bitches, the influence of some genetic factors during parturition has been reported. For example, the expression of oxytocin receptors in the endometrial and myometrial tissue increases during the late phase of the parturition [110], promoting normal delivery without the need for medical intervention. On the other hand, uterine inertia has been associated with an abnormal expression of γ-actin and myosin gene in the smooth muscle of the uterus, leading to dystocia in breeds such as Maltese, German Shepherd, Labrador Retriever, Beagle, and Boxer [111]. Foetal malposture and large foetuses are also considered causes of prolonged labour. It should be noted that obstetric interventions can increase stress and labour pain in addition to inducing more hemodynamic or vascular changes [112,113] (Table 2).

In felines, the breed has shown a strong correlation with dystocia incidence, with purebred cats such as British shorthairs showing a high incidence rate of 2.5. Likewise, 56% of complications during delivery require caesarean sections and intra- and postsurgical analgesia management in cats [114], in whom the administration of certain types of drugs requires care due to their biotransformation deficiencies.

In pigs, it is recognized that labour is a risky process for both the sow and the piglets and increases in the case of dystocic births due to prolonged parturitions [19]. The delivery in the sow has an average duration of 2.5 to 3 h, and when they last more than 3 h are considered potentially problematic and consequently more painful [90,115]. Maternal characteristics of the sow predisposing prolonged labour and potential pain are considered the breed, short gestation length, larger litter size, sow overweight, and parity; primiparous sows more prone to painful labour than pluriparous, among others. Higher birth weight and posterior presentation at birth are considered as foetal factors associated with dystocia [116,117,118,119,120,121,122,123,124,125,126,127] (Table 3). The widespread use of pigs in biomedical research is supported based on their physiological and anatomical similarities to humans, also large litters, and their large body size helps multiple samples collection. Pigs are omnivores with comparable digestive systems to humans [128].

Rats are widely used in biomedical research because of their physiological and anatomical similarity with humans. On the other hand, studies related to parturition, stress, and pain in other mammals such as mares, cows, and mice, have helped to understand neurobiological characteristics of parturition such as the parasympathetic predominance during the expulsion of the foetus. In case of stress and pain during the process can change towards a sympathetic influence with the consequent activation of sympathetic pathways that generate cortisol production, uterine atony, and prolongation of labour and dystocia [129,130]. However, Roussel et al. [131] report in ewes that moderate cortisol levels due to stress in the prenatal period (from 2.5 months of gestation) contribute to offspring with better birth weight, greater activity, and exploratory behaviours. Although pain during delivery has received little attention in cattle, the high prevalence of foetal-pelvic disproportions and the use of traction to remove the calf are events that involve pain and trauma, both for the mother and the calf generating rectovaginal ruptures that require pharmacological intervention for pain treatment [132].

## 6. Pain Assessment Scales

**Figure 2:**
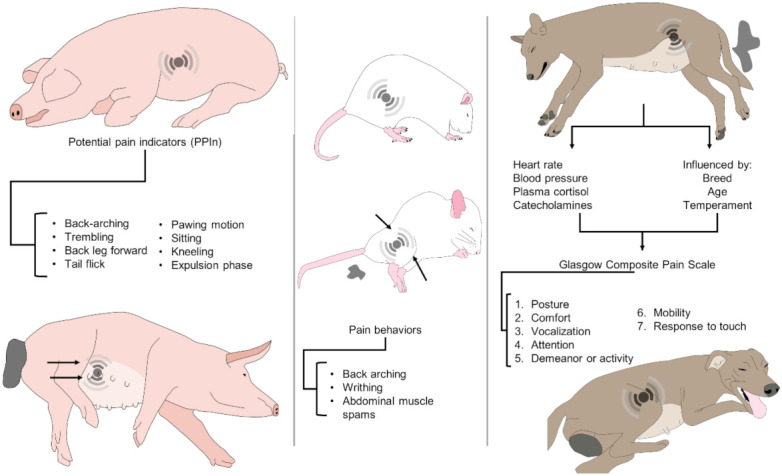
Clinical recognition of pain in sows, rats, and bitches. The recognition of pain during labor in these species can be done by observing altered postures or physiological parameters. In sows, potential pain indicators, such as a back-arching, one back leg forward, sitting, kneeling, trembling, among others, are modified behaviors during parturition. In rats, pain behaviors such as a compact posture, back-arching, writhing, and abdominal muscle contractions are pain signals. Lastly, the Glasgow Composite Scale (CMPS) in dogs uses 7 units in which posture, vocalizations, behavior, mobility, among others, are evaluated together with physiological parameters and plasma cortisol and catecholamine concentrations.

Pain is a personal subjective emotional state in humans and animals; therefore, it is complex to appreciate what each feel. Although it is feasible to assess pain in humans directly, using a degree scale measured by the subject [133], in non-verbal patients [134] and non-human animals, changes in physiological and behavioural parameters are used to assess the presence and severity of pain [135]. The sympathetic nervous system and the hypothalamic–pituitary–adrenal axis (HPA) mediate most physiological changes associated with painful stimuli. The sympathetic responses can be directly assessed by estimating the circulant catecholamines, adrenalin, and noradrenalin [136], or the resultant autonomic changes [137], body temperature, heart rate variability, and respiratory rate [138]. Glucocorticoids are used as biomarkers of stress, but also HPA changes in response to painful stimuli are commonly evaluated through the measure of glucocorticoid production, such as in rodents [139] (Table 4). Detection and evaluation of pain are crucial to improving welfare in a variety of contexts. Recently, in a review, it was recommended that any pain evaluation system should recognise that the pain event, at least in humans, has three elements: sensory-discriminatory, affective-motivational, and cognitive-evaluative, communicated between them to induce the experience of pain [17]. The experience of pain during labour results from processing multiple physiological, psychosocial, or behavioural factors (Table 5). Individual and subjective interpretation of labour pain by each person [5], and efforts to impartially and accurately estimate it in animals, are also especially challenging. In humans, the self-report is recognised as the “gold standard” for pain assessment, based on either oral or written communication [134]. However, it is described that verbal pain communication has limitations [140]. Several methods are described for assessing labour pain in women, including (a) the McGill Pain Questionnaire [MPQ], (b) The Short-Form MPQ, (c) Visual analogue scales [VAS], however, VAS are considered as the best system to measure pain [141] (Table 5). As mentioned above, while it is feasible a directly evaluate pain in humans using a grade scale scored by the subject [141], informative signs need to be analysed in animals to gather this information [133]. Indirect signs are helpful as indicators for evaluating pain in animals combine alterations in physiological and behavioural parameters [133,135]. Behavioural indicators to assess pain and labour pain across species are presented in Table 5 and Figure 2. Although objective measurements associated with acute pain are used in dogs (heart rate, blood pressure, and plasma cortisol and catecholamine levels), they are not reliable because stress, anxiety, and anaesthetics may influence them; therefore, pain assessment is mostly subjective and based on behavioural signs [133,142]. In the rat model for labour pain, measures include the activity of spinal neurons that receive afferents, using immunodetection of c-Fos protein, automated systems to detect pain behaviour and phase stretching behaviour [15]. In sows, a numerical pain score per animal is calculated by evaluating behavioural, clinical, and physiological patterns in a specific term [19]. However, behaviour is the most frequent indicator recommended for evaluating farrowing pain in sows; although intrauterine pressure may also be included [143] (Table 5).

A full evaluation of the pain being experienced is impossible since it is a subjective emotional state and our capacity to identify pain between species is even more challenging [17]. Nonetheless, several indicators of pain have been developed/identified for domestic species. However, they cannot be considered a “Golden Standard” for the presence of pain because detection of specific measures cannot be utilised to confirm or discard pain in non-verbal individuals [149].

Herskin and Di Giminiani [17] listed the seven most common indicators to evaluate pain in pigs (1) Motivational task, (2) Evoked behavioural responses, (3) Vocalisation, (4) Facial expressions, (5) Clinical, (6) Physiology and histopathological measures, (7) Pain scales. Similar indicators are used for most of the domestic species.

Ison et al. [91] evaluated behaviours as potential pain indicators in sows before, during, and post farrowing and two minutes before and after piglet deliveries. Behaviour evaluations included: back leg forward, tremble (shivering), back arch, paw (leg scraped in pawing motion), and tail-flick. All indicators were uncommon or absent pre-farrowing, highest during farrowing, and back leg forward, tremble, and back arches were higher at the immediate post-partum. Significant positive correlations between pain indicators during and post farrowing were found, including the back arch, tail-flick, and paw higher before than after a piglet birth and more frequent earlier in the birth order. However, the back leg forward and tremble did not differ before and after births, and the last increased with birth order. The authors concluded that these behaviours with consistent individual variation might be quantitatively associated with pain [91].

## 7. Managing Labour Pain

Pain relief in labour promotes maternal comfort and prevents the undesirable consequences of the catecholamine-mediated stress response [150]. Hence, the prevention and relief of pain through an accurate evaluation of this and the appropriate use of analgesics should be a priority for human medicine and animal science and production. The intention of pain relief during labour is to make the parturient relatively free of pain and also be able to participate in the childbirth experience. Preferably, side effects or associated risks for both the dam and newborn should be avoided. As presented in Table 6, there are several methods to relieve labour pain in humans and domestic animal species [21,150,151,152,153,154,155,156,157,158,159,160,161,162,163,164,165]. However, adverse effects have been reported, which could make them not ideal [151].

**Figure 3:**
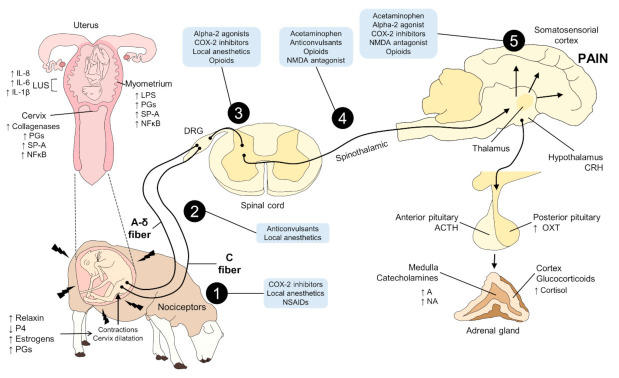
Sites of action of analgesics in the nociceptive pathway of parturition. During labour, hormonal factors such as increases in the concentrations of relaxin, estrogens, and PGs lead to cervical dilation and contractions. In the uterine tissue, LUS, myometrium, and cervix, different pro-inflammatory or degrading substances (such as collagenases) initiate an inflammatory response for the onset of softening and ripening the cervix. All of these factors activate peripheral nociceptors (A-δ and C fibers). These transduce the noxious stimulus into an electrical signal that is transmitted to the DRG of the spinal cord. Once in this structure, nociception modulation occurs, and drugs such as alpha-2 agonists, COX-2 inhibitors, local anesthetics, and opioids act at this site. These spinal neurons project to brain areas such as the thalamus or the somatosensory cortex, where pain perception occurs. Moreover, the activation of thalamic and hypothalamic areas leads to the secretion of other hormones such as ACTH or OXT. The adrenal gland contributes to the increase in circulating levels of A, NA, and cortisol, due to ACTH (cortisol) or by sympathetic influence (A, NA). Local anesthetics, NSAIDs, COX-2 inhibitors, opioids, acetaminophen, NMDA antagonist, and other analgesics act in each of the mentioned stages of the nociceptive pathway, depending on the nature of the drug. 1. transduction; 2: transmission; 3: modulation; 4: projection; 5: perception; A: adrenaline; ACTH: adrenocorticotropic hormone; CRH: corticotropin-releasing hormone; DRG: dorsal root ganglion; IL: interleukin; LPS: lipopolysaccharides; LUS: low uterine segment; NA: noradrenaline; NFκB: nuclear factor κB; NMDA: N-methyl-D-aspartate; NSAIDs: non-steroidal anti-inflammatory drugs; OXT: oxytocin; P4: progesterone; PG: prostaglandins; SP-A: surfactant protein A.

First of all, to choose the ideal analgesic to relieve pain during labour should consider: (1) provision of rapid pain relief in both the initial and secondary stage of labour without hazard or side effects to either the dam or the foetus; and (2) preservation of the mother’s motion capability and self-sufficiency during labour [69]. There are a wide variety of pharmacological techniques to alleviate pain, including oral pills, inhalation analgesia (i.e., nitrous oxide gas), intravenous and intramuscular opioids (pethidine or diamorphine), or narcotic drugs such as morphine, and various types of local (paracervical or pudendal block) and regional analgesia/anaesthesia (epidural or spinal anaesthetic and combined spinal and epidural (CSE) techniques) [159] (Figure 3). However, the criteria for choosing the analgesic method depend on the patient’s medical situation, the course of parturition and especially the in-place support availability [166]. Epidural analgesia (EA) is an effective method for pain management that adapts to the varied pain patterns experienced by female humans during childbirth [167]. Hence, analgesia can be achieved during the first stage of parturition by administering paracervical, paravertebral, or lumbar epidurals with local anaesthetics [13,153]. Although EA is considered the “gold standard”, it has been linked with a raised risk of assisted vaginal childbirth, maternal hypotension, motor blockade, maternal fever, urinary retention, oxytocin administration, the more prolonged second stage of parturition, and an increased risk of caesarean delivery due to foetal distress [21]. Therefore, a high number of studies compared the use of EA versus opioids, such as Remifentanil, which is suitable for administration through patient-controlled analgesia (PCA) [154,155]. However, opioids by parenteral administration are not as effective as EA and are not seen as an option when EA is available [157]. Although other drugs have been used, the EA remains the most efficient for releasing labour pain [4]. However, it should be highlighted that this method of analgesia is expensive and not available in many institutions [146]. In recent years, a growing interest has emerged in the combined spinal–epidural method (CSE) for labour analgesia, with the benefit of utilising lower doses of local anaesthetics and rapid analgesia [4,150,161,168]. Advancement in knowledge and developing a novel therapy for labour pain has been partially limited by the absence of an animal model for this kind of pain. This observation supposes that the physiology and pharmacology of labour pain vary from other kinds of pain. As research on various types of pain progresses, it is accepted that analgesia pharmacology significantly diverges [146]. The rat model is the most frequently used in biomedical research. It has been described that intrathecal administration of morphine (0.035–3.5 μg/h) approximately one day before delivery has an antinociceptive effect [146]. Despite the frequent use of conventional analgesic drugs like opioids, NSAIDs, and local anaesthetics to handle pain during a surgical procedure in laboratory animals [169], little is known about their efficacy and adverse effects on labour pain. Pharmacological management of analgesia at delivery (whelping) in bitches is only performed in the case of caesarean section (CS). The CS anaesthetic protocol model should produce good muscle relaxation, analgesia, and narcosis for optimal and secure surgical conditions for the bitch [170,171] and should not harm the vitality and survival of newborns [171,172]. The two classes of analgesics commonly used in veterinary patients are opioids and the nonsteroidal anti-inflammatory analgesics (NSAIDs) [163], despite analgesics and NSAIDs being problematic for CS in pregnant animals and humans [173,174]. The main reason is that opioids produce analgesia; however, they can reach the placenta and induce a critical central nervous system and respiratory depression in newborns. Nonetheless, Mathews [163] observes a consensus on the adequate safety of a 0.1 mg/kg single intravenous dose of meloxicam shortly after the parturition of the puppies. Nonsteroidal anti-inflammatory drugs are used to treat various conditions, such as post-operative pain [175].

Opioids are not registered for veterinary use in many countries, and therefore availability and accessibility of these drugs should be considered. The FDA in the US has approved some opioids specifically for use in animals, mainly in cats, dogs, horses, and wildlife. Due to the restricted products approved for use in animals, veterinarians use opioids approved for use in humans to control pain in their patients, but they must also follow regulations for extra-label use in animals, along with risk evaluations to assure that the benefits outweigh some risks [176].

Veterinary practitioners frequently use opioids to control pain associated with CS. However, the sensitivity to respiratory and CNS depression of opioids in newborn puppies due to the immaturity of their CNS is higher. Nevertheless, a small sublingual drop of naloxone in depressed puppies after delivery reverses the depressive effects of opioids [163]. While the use of analgesics has been widespread in companion animal medicine, some studies show that cattle often do not receive analgesia during painful procedures or conditions [177]. In pigs, the evidence of the adverse effects, on either the sow or piglets, caused by labour pain has increased the research interest on the use of analgesics around delivery; aiming to enhance/improve health, well-being, and productivity of both the sow and piglets [3,164,178,179]. Most studies are based on the analgesic administration after farrowing. Homedes et al. [180] and Sabaté et al. [178] reported the benefits of NSAIDs administration in the sow within 12 h after farrowing on the reduction of piglet mortality. Mainau et al. [179] reported an increase of the average daily weight gain and immunoglobulin uptake of low-birth-weight piglets (<1180 g) after the administration of meloxicam to the sow intramuscularly 90 min after farrowing.

Similarly, Viitasaari et al. [181] reported higher activity in young sows treated with ketoprofen for 3 days post-partum. In a recent study, 3 mg per kg of body weight or 1ml per 33 kg of body weight of ketoprofen was administered to gilts 90 min after the expulsion of the last piglet. Even though the study failed to prove clear production benefits of ketoprofen, high individual sow variation in piglet mortality, with some with regular performing and most of the piglet mortality happening in a low number of sows indicates a potential for targeted NSAID use [165]. In contrast, Mainau et al. [179] observed an improvement in average daily gain and immunoglobulin transfer in piglets, and reduced time sows spent lying down after oral administration of meloxicam to the sow as soon as possible at the beginning of the farrowing. Authors concluded that the oral meloxicam administration at the beginning of farrowing in pluriparous sows raised the serum IgG concentration of piglets and improved their preweaning growth [179]. The described effects of NSAIDs include reduction of post-partum oedema, pain, and inflammation and anti-endotoxic actions and help reduce preweaning mortality and piglet growth retardation from Post-Partum Dysgalactia Syndrome (PPDS).

## 8. Consequences of Pain

In female humans, the adverse outcomes of labour pain are assumed to arise primarily from changes in the maternal respiratory pattern and catecholamine-mediated stress response [5]. Brownridge [182] points out that the possible physiological consequences of critical parturition pain may involve increased oxygen consumption and hyperventilation, hypocarbia, and respiratory alkalosis. Furthermore, it may also involve autonomic stimulation and catecholamine release with gastric interference and hyperacidity, lipolysis, raised peripheral vascular resistance, cardiac output, blood pressure, decreased placental perfusion, and reduced uterine activity. These responses are hypothesised to produce maternal metabolic acidaemia, foetal acidosis, and dysfunctional labour. Such effects can be mostly harmless in the course of eutocic delivery [95]. However, pain that an individual is unable to control leads to increased release of catecholamines, reduced uterine contractions and the consequent dystocia, fatigue, foetal suffering, and postpartum posttraumatic stress disorder [5,96]. Several authors have reported that prolonged labour induces distress, fear and exhaustion, and the risk of damage, intrapartum and perinatal mortality. It also leads to increased use of oxytocin, higher frequency of CS and device use in vaginal delivery (i.e., vacuum or forceps), postpartum fever and reduced umbilical pH [95,183,184]. There are also descriptions that stress generated by parturition pain influences the diminished oxytocin level and prolonged labour. Beigi et al. [185] mention that fear of labour pain is one of the most important reasons female humans decide for a CS, even in pluriparous women [95,186]. Usually, a prolonged second phase during labour is associated with greater foetal and maternal complications like the atonic uterus, postpartum bleeding, and perineal trauma, operative vaginal delivery and episiotomy, increased risk of infection, foetal hypoxia, asphyxia, injuries, and perinatal mortality [104,187,188,189]. Women can experience different types of pain and discomfort following childbirth. It may involve surgical pain after CS, perineal pain after injury, or episiotomy through vaginal delivery; breastfeeding nipple pain and postpartum cramps pains associated with uterine involution. Ultimately, pain can also inhibit normal breastfeeding after delivery, diminishing the mother’s ability to care for the newborn and may threaten to establish a good-quality mother-baby relationship/interaction/bond [190]. The use of the rat model in pain investigation has played a pivotal role in recognising the types of nerves, receptors, ion channels, mediators, and biochemical pathways implicated in the origin, transmission, transduction, perception, and management of pain. However, there is no information about the consequences of labour pain in rats. Pregnancies in bitches are unique amongst domestic animal species because parturition is prolonged (up to 24–36 h), and newborns are very susceptible to environmental changes [191]. During parturition, physiological parameters change significantly due to pain, fear, and uterine contractions [192]. A high heart rate in the female dog results from a mixture of stress and myometrial contractions (uterine and abdominal) during labour [191]. As a response to pain, dam hyperventilation has unfavourable foetal outcomes. The consequences include respiratory alkalosis, compensatory progressive metabolic acidosis, becoming critical with the labour progress, and inducing foetal acidosis. Also, there are episodes of hypoventilation, leading to haemoglobin desaturation within contractions; and uterine vasoconstriction [191]. Stress hormones also drive to lipolysis caused by the liberation of free fatty acids (easily transferable through the placenta) and hyperglycaemia, which worsen foetal hypoxia. The above changes increase foetal acidosis, becoming progressively critical as parturition progress [193]. The passage from intrauterine to extrauterine life is usually accompanied by various degrees of hypoxia, which is very well tolerated by newborns [194]. However, if the labour time is prolonged, the survival of the newborn could be compromised. Birth is the most critical phase for neonates of all species and is associated with perinatal mortality and adverse effects on well-being [3,195,196]. In dogs, the stress associated with the birth process is usually an underlying cause of neonatal mortality, reported to be between 5% and 35% [196]. As we have presented, the process of farrowing in pigs, especially when dystocia occurs, has a high impact on sow’s well-being. Additionally, the stress of birth might impact the survival of newborns [195,196,197,198,199,200,201,202]. The presence of dystocia increases the risk of various conditions in the dam, including endometritis, vulvar secretion, placental retention, mastitis-metritis-agalactia syndrome, and impaired fertility [13,126,152,203]. On the other hand, piglets are particularly susceptible to intrapartum asphyxia [195,204]. Hypoxia and metabolic acidosis are consequences of asphyxia. They can cause severe effects on piglets’ health, vitality, and postnatal performance due to a reduced ability to reach a teat, consequently leading to an inadequate colostrum intake and passive immunity acquisition by the piglet [195]. On the other hand, acidosis can also cause hypothermia and reduce the survival of neonates [197,205]. An early indicator of foetal distress due to intrauterine hypoxia is meconium ejection into the amniotic sac, causing staining on the foetal skin and inhaling and lodging of meconium in severe cases the lungs [25,199,206]. On the other hand, the pain and stress associated with labour inhibit the release of oxytocin, leading to prolonged labour and reduction of colostrum and milk yield, consequently reducing nutritional and immunity supply to piglets [127,207]. Pain and stress of farrowing can also lead to restlessness and even aggressiveness in sows and bitches [208,209]. Insufficient milk production by sows with consequent malnutrition of the piglets can be directly responsible for 6 to 17% of the total pre-weaning mortality in commercial pig farms [210]. Metabolic and endocrine disorders in the sow, bacterial infections such as metritis (e.g., due to improper obstetric management), contribute to the decrease in prolactin secretion, and in primiparous sows, adequate suckling of their piglets is also inhibited [211].

## 9. Methods to Improve Pain Management during Parturition

Pain management in females during parturition and the controversy over the use of analgesics involves their dose-dependent tocolytic effect at this phase and its impact on uterine activity [212]. It is necessary to consider the pharmacokinetics of the drug and its affinity for the receptor. For example, a drug with a short plasma half-life may be an option to avoid the deleterious effects of pain during labor [213].

Likewise, pharmacokinetics helps to understand the effect that drugs may have on the neuroendocrine control of parturition, which has a fundamental physiological role. An example of this is morphine. This opioid can increase the plasma clearance rate of oxytocin [214], a hormone necessary during parturition. Therefore, drugs that have a higher affinity to its receptor or a prolonged plasma half-life could decrease the elimination time of this hormone.

On the other hand, an increase in uterine activity has been observed after the administration of amines [215], while drugs such as firocoxib do not affect uterine contractility [216]. These observations also suggest the implementation of analgesics with alternative pathways such as the inhibition of Ca2+ channels, such as gabapentinoids [217], NMDA receptor antagonists [213], or cannabinoid receptors [218], whose literature to date has not reported an alteration in uterine activity.

Other approaches that have been proposed to monitor and manage animals during the labour stage, such as motion-based technologies to infer parturition time even in wild species such as the Caribou (*Rangifer tarandus caribou*), are under development and have not yet reported conclusive data [219]. Machine-learning techniques to monitor wildlife and predict parturition have shown high rates of success (76% to 100% in wild ungulates [220]. Likewise, the monitoring of immune function, known as “immunity shift” has been proposed as a technique to evaluate health and immunity function, through rapid blood tests based on nanoparticles, and regularity of the physiological response in companion and farm animals [221].

## 10. Conclusions

Labour is a crucial moment for numerous species and also considered the most painful episode of females. Pain is fundamentally a psychophysiological phenomenon. However, unfortunately, pain in animals is not regularly recognised and is treated inappropriately. If parturition is a painful process identified in humans, it should also be considered painful in animals. To date, most publications on labour research focus on associated endocrine modifications, and there are fewer studies related to pain in the birth process. More robust comprehension of pain during farrowing in domestic animal species can generate new hypotheses and outcomes concerning its physiology and new pharmacological therapies, mainly concerning the relevance of analgesics and the welfare implications.

## Figures and Tables

**Table 2:** Factors associated with dystocia, prolonged farrowing and pain in the bitch.

Factor That Increases Labour Pain	References
**Maternal characteristics:** Prolonged labours Primary uterine inertia. Caused by numerous factors including sepsis, disease, age-related abnormalities, or a genetic disability of myometrial contractility. Age of the bitch. Older age (older than 6 years) predisposes to more single-pup pregnancies, uterine inertia, and prolonged parturition compared with the younger. Litter size. Pregnancies with few foetuses do not allow the start of labour properly. Low numbers of foetuses (1–2 puppies) represented 21.5% of dystocia. Breed. Obstruction due to a short pelvic channel is common in brachycephalic and achondroplastic breeds. It is also challenging to give birth to toy dogs, prone to small litters and large foetuses. Obstructive dystocia. Due to several factors such as uterine torsion, uterine rupture, inguinal hernia, and abnormalities of the soft tissues of the vagina or vulva. Hypocalcaemia. **Foetal characteristics:** Foetal Malposture. Large foetus. Pregnancies with a large foetus, such as, a single puppy, gestational diabetes; or foetal anomalies like ana sarca (“water puppy”) or a foetal monstrosity.	[62,108,113]

**Table 3:** Factors associated with dystocia, prolonged farrowing and pain in the sow.

Factor That Increases Labour Pain	References
**Maternal characteristics:** Duration of farrowing (more than 3 h): Breed. Short gestation length. Increased litter size. Increased number of stillborns. Sows with overweight. Constipation. Lack of exercise. Parturitions: primiparous sows go through a more painful labour process than pluriparous. **Foetal characteristics:** Higher birth weight. Birth order. Presentation at birth. This is a controversial issue; however, some reports relate the posterior presentation of piglets at birth with the increase of the delivery duration. **Others:** Misuse in medication control at labour with the use of ecbolics (such as oxytocin). Excessive obstetric manipulation.	[1,19,116,117,120]

**Table 4:** Multi-parametric scales for assessing labour pain (physiological indicators) in human Obstetric and Veterinary Medicine.

Responses	Variables and Scales	References
Sympathetic-Adreno-Medullary System	Cardiac rate. Rectal temperature. Respiratory rate. Blood pressure.	[135,138]
Hypothalamic Pituitary Adrenocortical System	Cortisol. Adrenalin. Noradrenalin. β-endorphin (β-end). Met-encephalin. Oxytocin. C-reactive protein (CRP) and cytokines. Duration of labour. Number of stillbirths (only for animals).	[135,136,139]

**Table 5:** Multi-parametric scales for assessing labour pain (behavioural indicators).

Species	Variables and Scales	References
Women	(a) McGill Pain Questionnaire [MPQ], (b) The Short-Form MPQ, (c) Visual analogue scales [VAS]. Verbal rating scales, or simple ordinal scales.	[5,134,141]
Dog	The behavioural representation of pain is species-specific and affected by age, breed, temperament, and additional stressors, including anxiety or fear; hence, it is necessary to identify the normal behaviour of the bitch. The composite measure pain scale (CMPS) has been described for use in dogs with acute pain, based on seven categories: (1) posture, (2) comfort, (3) vocalization, (4) attention to wound, (5) demeanour, (6) mobility, and (7) response to touch.	[16,23,133,142,144]
Rat	Activity of spinal neurons that receive afferents, using immunodetection of c-Fos protein, an indirect indicator of harmfully activated spinal neurons. Labour induced the expression of neuronal c-Fos in segments T12-S2 of the spinal cord one hour following the delivery of the first pup. Automated systems for the detection of pain behavior. General activities, including food and water consumption, rearing (upright exploration), and chest and head grooming, are evaluated to determine spontaneous behaviour during labour. Phase stretching behaviour during labour included: crushing (an asymmetrical contraction of the lower body and limbs), sidelong contraction (an asymmetric contraction of the lower body and limbs), lengthening (stretching of the abdomen and the four limbs) and phasic humpback posture. Maternal care activities included building nests, licking pups, and eating the placenta.	[15,145,146]
Sow	Numerical pain score per animal is calculated by evaluating behavioural, clinical, and physiological patterns in a specific term. Observation of certain behaviours as potential pain indicators (PPIn) in sows before, during and after farrowing: hind leg forward (in a side-lying posture, the back leg is pulled ahead and/or in towards the body), back arch (in a side-lying position, one or both legs turn rigid and are pushed aside from the body and/or inwards towards the centre, creating a curvature in the back). Putative indicators of pain used to evaluate the pain behaviour in the periparturient sow. Also, it coincides with an activity shift from nest buildings to passivity, an increase of the myometrial electrical activity, and the increment of oxytocin levels before the beginning of the ejection of piglets. Postural changes considered: stand (upright, with all feet on the floor), sit (front legs straight and back end down on the floor), lie lateral (lying on one side with udder exposed), kneel (front knees on the ground, with hinds legs straight), lie ventral (lying with the udder on the floor), tremble (visibly shaking as if shivering when in a lateral lying position), and others; tail-flick (tail is moved rapidly up and down), paw (in a lateral lying position, the front leg scraped in a pawing motion), piglet delivery (piglet completely ejected from the dam).	[3,74,147,148]

**Table 6:** Pharmacological analgesia for labour pain in humans and domestic animals.

Species	Route	Drug	Observations	References
Women	Inhalational analgesia (reduce pain perception).	1. Entonox. 2. Isoflurane. 3. Desflurane. 4. Sevoflurane.	Use limited to developed countries.	[13,21,150]
	Parenteral opioids (reduce pain perception).	1. Pethidine. 2. Morphine. 3. Diamorphine. 4. Fentanyl. 5. Remifentanil. 6. Meperidine.	Adverse effects have been observed.	[13,21,150,153,154,155,156,157,158,159]
	Regional analgesia (reduce pain transmission).	1. Epidural. 2. Combined spinal-epidural (CSE). Lidocaine. Xilacina. Levobupivacaine. Bupivacaine.	Epidural analgesia is the “gold standard” for labour analgesia.	[13,153,158,160,161]
Bitch	Intravenous Intramuscular.	1. Opioids (Methadone, Morphine) 2. AINEs (Meloxicam).	Use limited to caesarean section.	[162,163]
Rat	Intrathecal.	Morphine.	Used in research model for labour pain.	[146]
Sow	Intramuscular, Oral.	Ketoprofen. Meloxicam.		[3,164,165]
